# Cervical Cancer Imaging Features Associated With *ADRB1* as a Risk Factor for Cerebral Neurovascular Metastases

**DOI:** 10.3389/fneur.2022.905761

**Published:** 2022-07-12

**Authors:** Xingju Zheng, Shilin Xu, JiaYing Wu

**Affiliations:** ^1^Department of Radiology, Guizhou Provincial People's Hospital, Guiyang, China; ^2^Department of Oncology, Xichang People's Hospital, Liangshan High-Tech Tumor Hospital, Xichang, China; ^3^Department of Gynaecology and Obstetrics, Zhejiang Xinda Hospital, Huzhou, China

**Keywords:** cervical cancer, brain metastasis, neurovascular, clinical prediction model, radiomics, *ADRB1*, biomarkers

## Abstract

Bioinformatics tools are used to create a clinical prediction model for cervical cancer metastasis and to investigate the neurovascular-related genes that are involved in brain metastasis of cervical cancer. One hundred eighteen patients with cervical cancer were divided into two groups based on the presence or absence of metastases, and the clinical data and imaging findings of the two groups were compared retrospectively. The nomogram-based model was successfully constructed by taking into account four clinical characteristics (age, stage, N, and T) as well as one imaging characteristic (original_glszm_GrayLevelVariance Rad-score). In patients with cervical cancer, headaches and vomiting were more often reported in the brain metastasis group than in the other metastasis groups. According to the TCGA data, mRNA differential gene expression analysis of patients with cervical cancer revealed an increase in the expression of neurovascular-related gene Adrenoceptor Beta 1 (*ADRB1*) in the brain metastasis group. An analysis of the correlation between imaging features and *ADRB1* expression revealed that *ADRB1* expression was significantly higher in the low Rad-score group compared with the high Rad-score group (*P* = 0.025). Therefore, *ADRB1* expression in cervical cancer was correlated with imaging features and was associated as a risk factor for cerebral neurovascular metastases. This study developed a nomogram prediction model for cervical cancer metastasis using age, stage, N, T and original_glszm_GrayLevelVariance. As a risk factor associated with the development of cerebral neurovascular metastases of cervical cancer, *ADRB1* expression was significantly higher in brain metastases from cervical cancer.

## Introduction

The fourth most prevalent cancer among women worldwide is cervical cancer, a common gynecologic malignancy (1). Approximately 570,000 new cases of cervical cancer were reported in 2018, representing 3.15% of all malignant tumor cases. Furthermore, approximately 310,000 cervical cancer-related deaths were reported in 2018, representing 3.26% of all malignant tumor deaths (1, 2). Cancer metastasis is a major cause of poor prognosis in cervical cancer (3–6). The five-year survival rate for cervical cancer patients with metastasis is only 30–60%, which is significantly lower than that for those without metastasis (7, 8). Cervical cancer metastasis involves complex molecular mechanisms (9). The lack of effective measures to prevent or treat cervical cancer metastasis is attributable to the fact that the mechanism of cervical cancer metastasis remains unclear.

Cervical cancer metastasis occurs mainly via the lymphatic route (10). The most common sites of distant metastases are the lungs, bones, and liver, in order of priority (11). It is rare for cervical cancer to spread to the central nervous system, and very little research has been conducted on this subject (12, 13). When diagnosed with brain metastases, cervical cancer patients typically have a poor prognosis; the median survival period from the time of diagnosis until death is 2–3 months (13). As of now, there is no reliable way to predict early whether cervical cancer will spread to the neurovascular system.

Known as an emerging method of image analysis, radiomics can extract quantitative features from medical images that describe biological information, such as tumor characteristics and heterogeneity, which can serve to guide clinical planning (14). In patients with esophageal cancer, radiomics also proved to be effective for predicting T and N stages as well as lymph node metastasis (15–17). Based on the results of the radiomics study, the model can improve the diagnostic accuracy of differentiating benign and malignant cartilage tumors, as well as benign and low-grade malignant chondrosarcomas (18). An analysis conducted by Zhao et al. of diffusion-weighted images of osteosarcoma was retrospectively screened for four clinical features and eight imaging features, and a combined feature model was constructed for both characteristics, displaying good performance (19). Thus, it is possible to assess a tumor and predict its progression by combining imaging and clinical data. Moreover, imaging analysis is useful in determining the malignant and benign classification and staging of ovarian tumors, monitoring treatment outcomes in patients with metastatic ovarian tumors earlier in the course of treatment, and providing high-precision survival assessment for patients with ovarian tumors (20, 21). Consequently, radiomics is an important tool for predicting tumorigenesis, metastasis, and prognosis.

Radiomics has made significant progress in recent years when it comes to detecting and localizing brain metastases, making a differential diagnosis with other primary brain tumors, and determining the prognosis of the patient. An algorithm based on multi-sequence magnetic resonance imaging (MRI) for example, is capable of automatically detecting and segmenting brain metastases with great precision (22). The best results of glioblastoma multiforme and metastasis differentiation can be obtained by applying an Support Vector Machine (SVM) classifier based on enhanced T1-weighted images (T1W1) radiomics (23). Furthermore, the enhanced T1W1 imaging feature – “zone percentage” is an independent prognostic factor for local tumor control in non-small cell lung cancer brain metastases (24). Researchers from Shen et al. performed a retrospective study using computed tomography (CT) images of patients with bones metastases from lung adenocarcinoma who had genetically confirmed epithelial growth factor receptor (*EGFR*) mutation status. Three features were associated with *EGFR* mutation status (25). Yu et al. demonstrated that high-throughput MRI imaging features extracted from conventional T2 fluid attenuation inversion recovery (T2-FLAIR) images were highly correlated with isocitrate dehydrogenase genotype 1 (*IDH1*) (26). Furthermore, the conventional sequence-based fusion imaging model constructed by Jiang et al. accurately predicted the methylation status of O6-methylguanine-DNA methyltransferase (MGMT) in low-grade gliomas (27). Hence, imaging features have a potential superior predictive efficacy in predicting tumor genotypes (28).

Several studies have reported that neurovascular factors are involved in the development and metastasis of several malignancies (29, 30). The adrenergic receptor gene, a neurovascular-related factor, encodes the adrenergic receptor.

β-adrenergic receptor blockers have the ability to reduce the risk of head and neck cancer, as well as esophageal, gastric, colorectal, and prostate cancer (31, 32). Catecholamines promote ADRB2-dependent pancreatic ductal adenocarcinoma and neurotrophic factor secretion, which is critical to tumor development (33). The induction of angiogenesis by brain endothelial cells was shown to be crucial for the early survival of brain metastatic cells (34). Further, patients with lung cancer with a positive *VEGFC* expression were significantly more likely to develop brain metastases than those with a negative *VEGFC* expression (35). Accordingly, neurovascular-related genes are involved in brain metastasis in cervical cancer, and it may be possible to predict neurovascular-related genes that are associated with brain metastasis by imaging features.

In recent years, bioinformatics has gained significant attention in several fields for its role in exploring disease mechanisms (36–41). The objective of this study was to identify early risk factors and potential targets from an imaging and genomics perspective for patients with brain metastases from cervical cancer in order to improve prognoses. By combining radiomics data and clinical information, a prediction model reflecting the risk of cervical cancer metastasis was constructed in this study that screened neurovascular genes with brain metastases related to cervical cancer.

## Methods

### Data Collection

Patients treated at the Guizhou Provincial People's Hospital between January 2010 and January 2021 for CT examinations and a biopsy or surgical pathology that confirmed cervical cancer were included in the study. Inclusion criteria were as follows: I. an accurate pathological diagnosis; II. archived CT examination data; III. comprehensive medical and follow-up records. The following exclusion criteria were applied: I. incomplete clinical information; II. concurrent presence of primary malignant tumors at other sites; III. inadequate image quality. A total of 118 patients with cervical cancer participated in the study. The study was approved by the Ethics Committee of Guizhou Provincial People's Hospital.

### Acquisition and Differential Testing of Neurovascular-Related Gene Expression Data

From the TCGA database, clinical information and transcript information were downloaded for two patients with brain metastases and 15 patients with extracerebral metastases from cervical cancer. The RNA transcription data were log-transformed and de-batched. In total, 469 genes related to neural or vascular function were identified through a literature review using PubMed, Web of Science, Google Scholar, and GeneCards databases. These genes regulate the interaction between nerves and blood vessels. The R package “limma” (version 3.50) was used to conduct gene expression analyses of groups with brain metastases and those with extracerebral metastases. Genes with false discovery rate < 0.05 and |log2 FoldChange| >1 were filtered for subsequent analysis.

### Image Processing and Radiomics Feature Extraction

GE 16-row spiral CT scanners were used to acquire the imaging data of all patients. All patients were injected with 500 ml of pantethine (Xi'an Hanfeng Pharmaceutical Co., Ltd.), using a 5 mm needle, 30 min before scanning. CT images of patients were read and processed in Digital Imaging and Communications in Medicine (DICOM) format. ITK-SNAP (version 3.8.0; https://www.itksnap.org) was used for image segmentation, whereas the region of interest (ROI) was manually outlined, layer by layer, along the tumor edge. Finally, the segmented images were subjected to radiomics feature extraction using the Pyradiomics (version 3.0.1) package. Spearman's correlation analysis was used to assess the correlation between the radiomics features. For radiomics features with correlation coefficients > 0.85, only one was retained, thus minimizing redundant features.

### Logistic Regression Analyses

Clinical and imaging characteristics were subjected to univariate logistics regression analysis, and characteristic variables with *P* < 0.05 were retained. Based on previous studies, we constructed a nomogram prediction model (40, 42–44). The retained imaging and clinical characteristics were included in the multivariate logistics regression analysis together, and characteristics with *P* < 0.1 were retained. The odds ratio (OR) and 95% confidence interval (95% CI) for each variable was determined separately. The receiver operating characteristic curve (ROC) was used to assess the sensitivity and specificity of continuous variables for predicting tumor metastasis.

### Nomogram Construction and Clinical Application Validation

A multivariate logistics model was developed using the retained clinical and imaging characteristics. The entire dataset was divided into training and test sets in the ratio of 7:3, and the predictive performance of the model was quantitatively evaluated using the area under the curve (AUC) of the ROC. Decision curve analysis (DCA) calculated the net yield at different threshold probabilities to assess the utility and safety of the clinical prediction model. To facilitate clinical use, “RMS” (version 6.2) was used to visualize this combined multivariate model by constructing a nomogram. Predictors in the nomogram included select clinical and radiomics features. Calibration curves were used to assess the concurrence between predicted probabilities and actual outcomes.

### Gene Set Enrichment Analysis

GSEA was performed to identify target neurovascular-related gene pathways using the RNA transcript dataset of patients with cervical cancer from TCGA database (45). The R package cluster profile (version 4.0) was used for GSEA analysis (46). The reference gene set “c2.cp.v7.2.symbols.GMT [Curated]” (MSigDB Collections, https://www.gsea-msigdb.org/gsea/msigdb/) was used for functional pathway annotation. The significant difference was adjusted to *P* < 0.05 as the cut-off criterion.

### Quantitative Reverse Transcription Polymerase Chain Reaction

Patient tumor tissue samples were analyzed using qRT-PCR to explore the relationship between imaging features and gene expression. TRIzol reagent (Invitrogen) kits were used to extract total RNAs from the samples. According to the manufacturer's instructions, stem-loop antisense primer mix and AMV transcriptase (TaKaRa, China) kits were used to reverse transcribe these total RNAs into cDNAs. Finally, qRT-PCR was performed in the ABI 7500 real-time PCR system (Applied Biosystems, CA, USA) using an SYBR QPCR kit (Toyobo, Osaka, Japan). The primer sequences used are as follows: for *ADRB1*, forward: 5′-TCTACGTGCCCCTGTGCAT-3′ and reverse: 5′-TCGATCTTCTTCACCTGCTTCTG-3′; for *GAPDH*, forward: 5′-AAGAAGGTGGTGAAGCAGGC-3′ and reverse: 5′-TCCACCACCCAGTTGCTGTA-3′; for GAPDH, forward: 5′-AAGAAGGTGGTGAAGCAGGC-3′ and reverse: 5′-TCCACCACCCAGTTGCTGTA-3′ (47). *GAPDH* was used as a reference to normalize the same gene expressions between samples. The 2^−Δ*ΔCt*^ method was used to calculate the relative expression of the target gene. Gene expression was further normalized between samples before subsequent analysis.

### Statistical Analysis

Demographic variables were divided into categorical and continuous variables, with Pearson's chi-square or Fisher's exact tests being used for categorical variables and Wilcoxon rank sum tests for continuous variables. Statistical analysis was performed using R software (version 4.0.2), and a two-tailed *P* < 0.05 was considered a statistically significant difference.

## Results

### Demographic Characteristics and Imaging Features of the Patients

**Figure 1:**
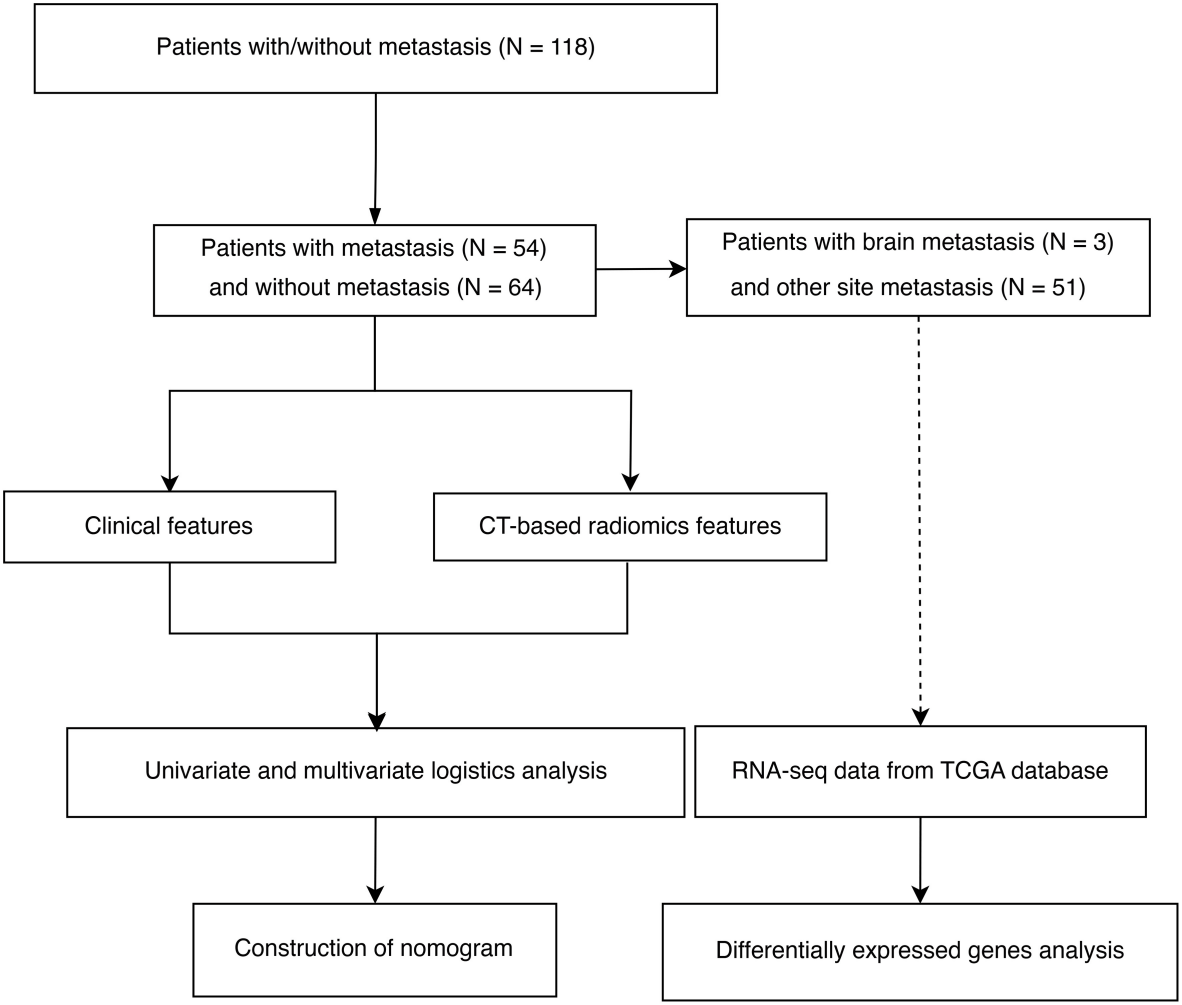
Flowchart showing the inclusion criteria and study process of selecting patients with cervical cancer.

**Figure 2:**
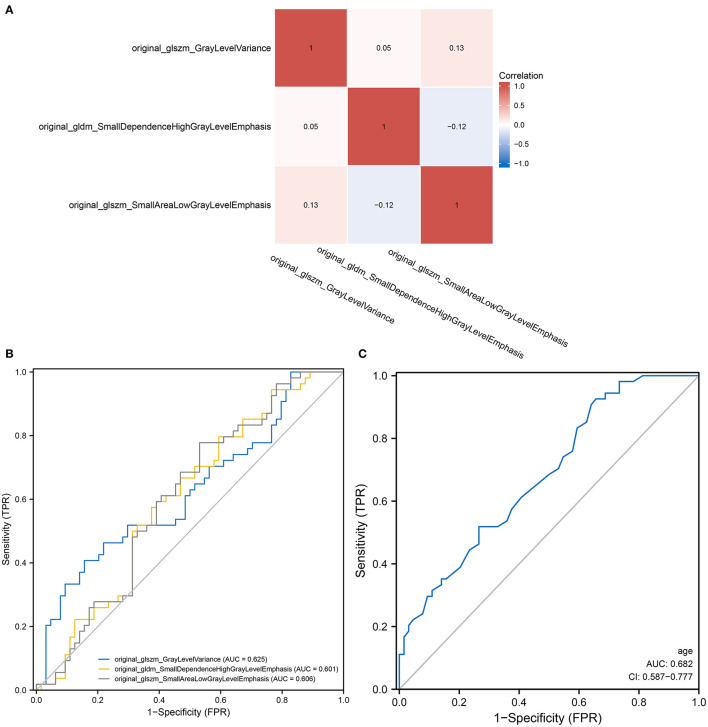
Radiomics and the predictive power of age in predicting tumor metastasis. **(A)** A heat map demonstrating low correlation between the three screened radiomics features; **(B)** A heat map demonstrating low correlation between the three screened radiomics features; **(C)** A ROC curve showing the ability of univariate logistic regression models of age to predict tumor metastasis.

The flow chart of patient inclusion and data sources in this study are shown in Figure 1. The clinical characteristics and imaging variables of 118 patients with cervical cancer are presented in Table 1. Older patients showed a higher incidence of tumor metastasis than younger patients (*p* = 0.022). Additionally, a statistical difference was observed in stage, metastasis, T, N, surgery and radiation between patients with and without metastasis (*P* < 0.05). Patients with metastasis had a higher stage, larger T and N grades and lower surgery and radiation therapy rates than those without metastasis. Analyses of univariate regression and correlation were performed on the features extracted from the enhanced CT images, identifying three most critical variables, namely original_glszm_GrayLevelVariance, original_gldm_SmallDependenceHighGrayLevelEmphasis and original_glszm_SmallAreaLowGrayLevelEmphasis. The correlation heatmap of these three features is shown in Figure 2A. Based on the above analysis, we identified the main imaging features of metastases from cervical cancer.

**Table 1:** Demographic and clinical characteristics of patients with or without metastasis.

**Variables**	**Level**	**Metastasis**		
		**No**	**Yes**	***P*-value**
		**(*N* = 64)**	**(*N* = 54)**	
Age [meidan (IQR)]		47.5 (35.75, 53.25)	50 (42.25, 60)	0.022*
Height [meidan (IQR)]		160 (157, 162)	159 (157, 163.5)	0.363
Weight [meidan (IQR)]		66 (56, 73.25)	69 (57.25, 79.75)	0.303
Marital status [N (%)]	Divorced	6 (9.4%)	3 (5.6%)	0.152
	Married	48 (75%)	48 (88.9%)	
	Unmarried	10 (15.6%)	3 (5.6%)	
Stage [N (%)]	I	41 (64.1%)	11 (20.4%)	<0.001***
	II	17 (26.6%)	15 (27.8%)	
	III	6 (9.4%)	15 (27.8%)	
	IV	0 (0%)	13 (24.1%)	
Histology [N (%)]	Adenosquamous	4 (6.2%)	5 (9.3%)	0.261
	Mucinous Adenocarcinoma	2 (3.1%)	5 (9.3%)	
	Squamous Cell Carcinoma	58 (90.6%)	44 (81.5%)	
Metastasis site [*N* (%)]	Bone	0 (0%)	9 (16.7%)	<0.001***
	Brain	0 (0%)	3 (5.6%)	
	Liver	0 (0%)	8 (14.8%)	
	Lung	0 (0%)	22 (40.7%)	
	No	64 (100%)	0 (0%)	
	Others	0 (0%)	12 (22.2%)	
N [*N* (%)]	N0	46 (71.9%)	9 (16.7%)	<0.001***
	N1	18 (28.1%)	45 (83.3%)	
T [*N* (%)]	T1	37 (57.8%)	11 (20.4%)	<0.001***
	T2	19 (29.7%)	18 (33.3%)	
	T3	7 (10.9%)	11 (20.4%)	
	T4	1 (1.6%)	14 (25.9%)	
Surgery [*N* (%)]	No	28 (43.8%)	42 (77.8%)	<0.001***
	Yes	36 (56.2%)	12 (22.2%)	
Radiation [*N* (%)]	No	23 (35.9%)	7 (13%)	0.008**
	Yes	41 (64.1%)	47 (87%)	

^*^ P <0.05,

^**^ P <0.01,

^***^ P <0.001.

### Variable Screening for Univariate and Multivariate Logistic Regression Analysis

**Figure 3:**
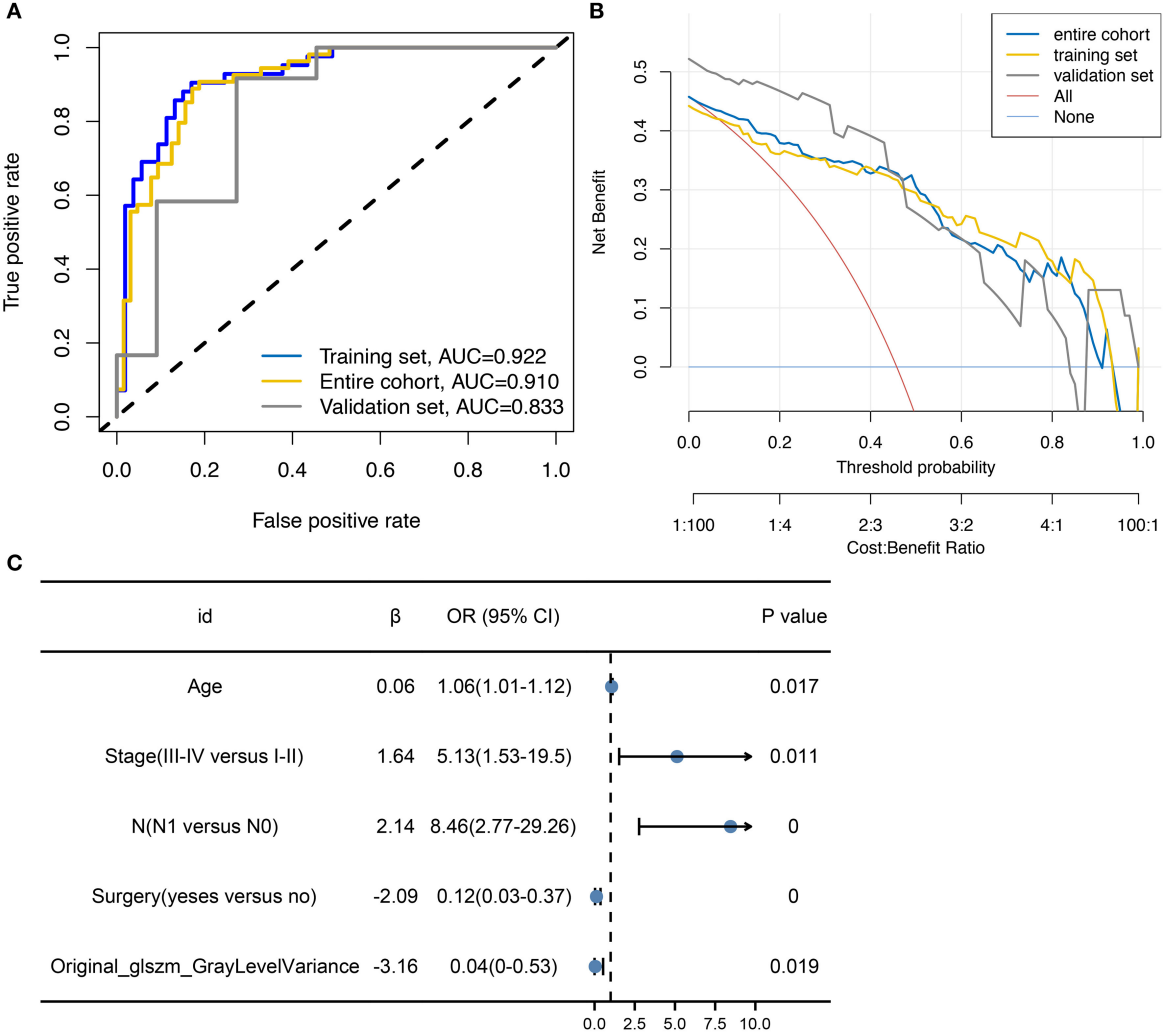
Construction of multifactor models with variable weights. **(A)** ROC curve showing the predictive performance of models based on clinical data and imaging features. **(B)** DCA showing the wide range of clinical utility and safety associated with the built prediction model. **(C)** Forest plot displaying the relative weights and values of the variables included in the model.

Logistic models are commonly used to screen for risk factors for clinical outcomes. The predictors associated with tumor metastasis based on univariate and multivariate logistic regression models are shown in Table 2. Based on the filtering conditions of univariate analysis, age, stage, N, T, surgery, radiation, original_glszm_GrayLevelVariance, original_gldm_SmallDependenceHighGrayLevelEmphasis and original_glszm_SmallAreaLowGrayLevelEmphasis were able to predict tumor metastasis in patients with cervical cancer (*P* < 0.05). The AUCs for these three radiomics features were 0.625, 0.601, and 0.606 according to the univariate analysis, which means these features can serve as good predictors of cervical cancer (Figure 2B). Further multivariate logistic regression analysis showed that age, stage and N were independent predictors of tumor metastasis (*P* < 0.05). According to Figure 2C, the AUC on the prediction model constructed based on age was 0.682 (0.587–0.777). Based on predefined filtering conditions, age, stage, N, surgery, and original_glszm_GrayLevelVariance have been incorporated into a multivariate logistic regression model in order to construct a clinical prediction model. In the training, validation, and overall groups, the AUCs of this predictive model were, respectively 0.922, 0.833, and 0.910 (Figure 3A). Accordingly, the predictive efficacy and accuracy of the model was enhanced by the inclusion of clinical and imaging factors together. Based on DCA of the training, validation and overall groups shown in Figure 3B, the model appears to be user-friendly and has a wide range of clinical applicability. Figure 3C depicts the weights assigned to each variable in the model, with the imaging features having the greatest weight (Table 3).

**Table 2:** Univariate and multivariate logistics regression analysis.

**Variables**	**Univariate logistic regression**			**Multivariate logistic regression**		
	**OR**	**95% CI**	***P*-value**	**OR**	**95% CI**	***P*-value**
Age*	1.07	1.03, 1.1	<0.001	1.07	1.02, 1.14	0.014
Height*	0.99	0.94, 1.05	0.758	NA	NA	NA
Weight*	1.02	0.99, 1.05	0.325	NA	NA	NA
**Marital status (Married)**						
Unmarried	0.3	0.06, 1.05	0.081	NA	NA	NA
Divorced	0.5	0.1, 2.01	0.346	NA	NA	NA
**Stage (I-II)**						
III-IV	10.41	4.07, 30.65	<0.001	6.65	1.67, 32.56	0.011
**Histology (adenosquamous)**						
Mucinous adenocarcinoma	2	0.26, 19.77	0.518	NA	NA	NA
Squamous cell carcinoma	0.61	0.14, 2.42	0.476	NA	NA	NA
**N (N0)**						
N1	12.78	5.4, 33.04	<0.001	7.01	1.93, 28.97	0.004
**T (T1)**						
T2	3.19	1.27, 8.31	0.015	1.91	0.45, 8.16	0.375
T3	6.17	1.92, 21.77	0.003	0.48	0.06, 3.27	0.459
T4	23.55	5.53, 165.7	<0.001	2.11	0.2, 34.99	0.558
**Surgery (No)**						
Yes	0.22	0.1, 0.49	<0.001	0.13	0.03, 0.43	0.002
**Radiation (No)**						
Yes	3.77	1.53, 10.33	0.006	1.42	0.33, 6.19	0.634
Original_glszm_GrayLevelVariance*	0.1	0.02, 0.6	0.013	0.08	0, 1.24	0.081
Original_gldm_SmallDependenceHighGrayLevelEmphasis*	0.13	0.02, 0.69	0.022	0.21	0.01, 3.2	0.276
Original_glszm_SmallAreaLowGrayLevelEmphasis*	0.13	0.02, 0.64	0.016	0.25	0.02, 2.31	0.241

^*^Continuous variable.

**Table 3:** Prediction factors for nomogram.

**Viariable**	**Prediction model**		
	**β**	**OR (95% CI)**	***P*-value**
(Intercept)	−2.19	0.11 (0.01–1.53)	0.11
Age	0.06	1.06 (1.01–1.12)	0.02
Stage III-IV	1.64	5.13 (1.53–19.50)	0.01
N(N1)	2.14	8.46 (2.77–29.26)	0.00
Surgery (Yes)	−2.09	0.12 (0.03–0.37)	0.00
original_glszm_GrayLevelVariance	−3.16	0.04 (0.00–0.53)	0.02

### Nomogram Construction for Predicting Tumor Metastasis

**Figure 4:**
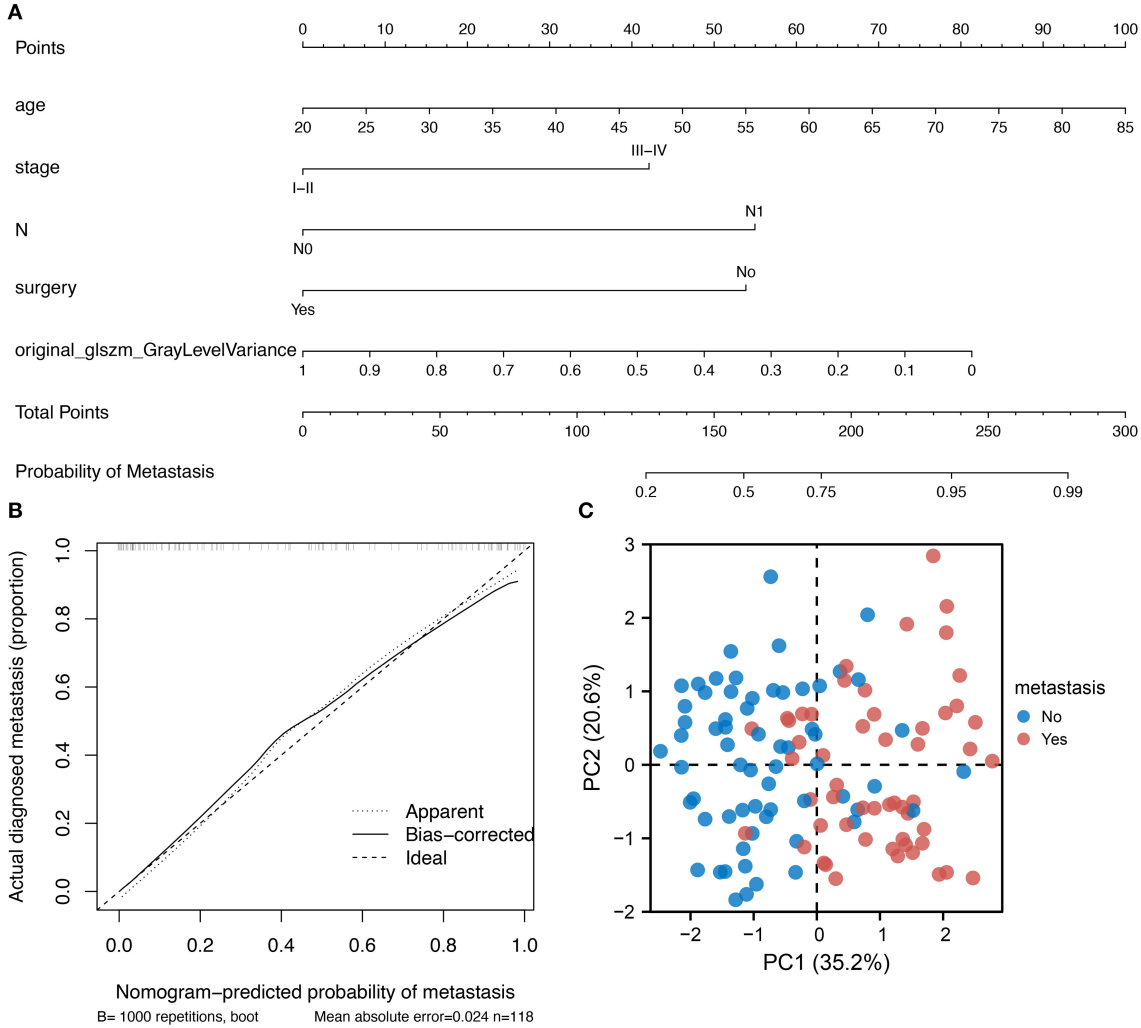
Nomogram development and clinical validation. **(A)** A nomogram was constructed using the multi-factor logistic regression model; **(B)** Calibration plot showing a good fit between the true and predicted values; **(C)** PCA showing the variables incorporated into the model could distinguish between cervical cancer patients with metastasis.

Based on the regression coefficients of each variable in the constructed multivariate logistic regression model, the nomogram of the clinical prediction model was plotted (Figure 4A). The nomogram consists of a series of selected variables and a score corresponding to each featured variable. By calculating the total score of all features in the nomogram, the likelihood of tumor metastasis can be determined. It is evident in Figure 4B that there is a high degree of consistency between the predicted and true values for patients in the multivariate prediction model. Principal component analysis (PCA) reveals that the variables incorporated in the model show significant differences between patients with cervical cancer with and without metastasis (Figure 4C). These results suggest that this cervical cancer metastatic prediction model performs well.

### Comparison of the Brain and Non-brain Metastasis Groups

**Figure 5:**
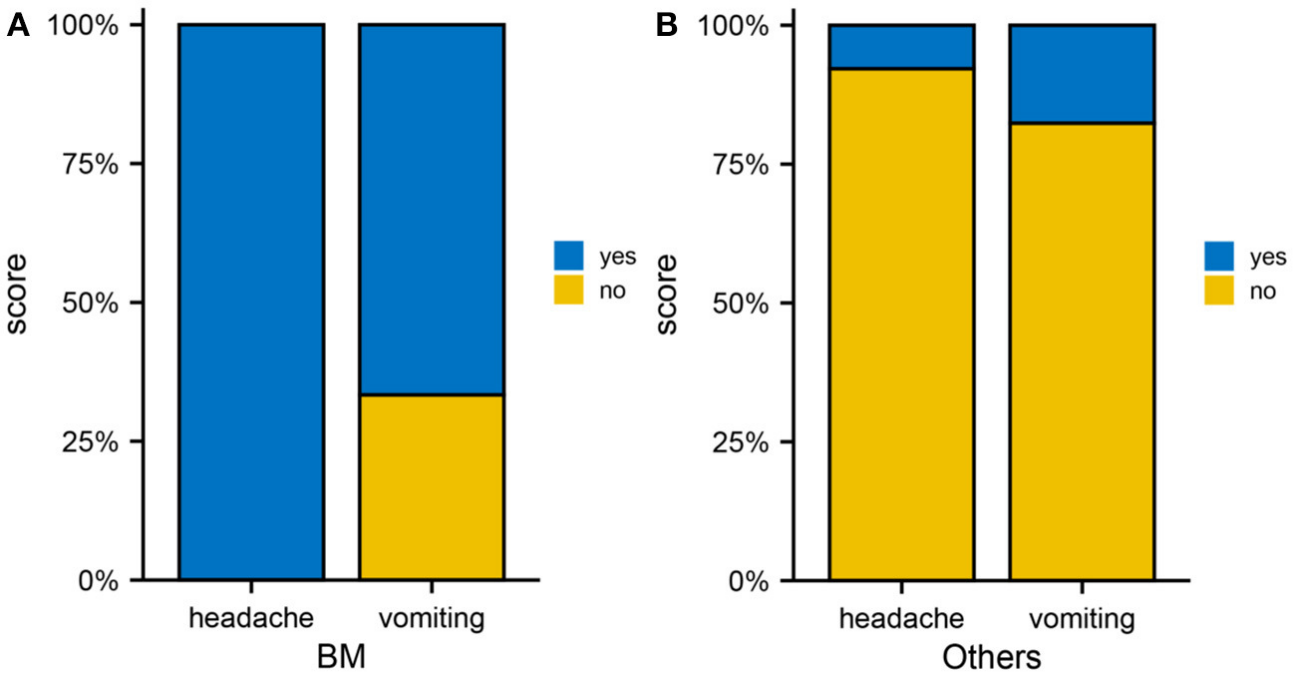
Brain metastases from cervical cancer. **(A)** Occurrence rate of headache as well as vomiting in the brain metastasis group. **(B)** Occurrence rate of headache and vomiting in the non-brain metastasis group.

An examination of clinical differences between patients with brain metastases and those with non-brain metastases was performed to determine the relationship between metastases, neurological and vascular effects. Among the brain metastases group, the incidence of headache and vomiting was 100 and 66.7%, respectively (Figure 5A), whereas among the metastases group, the incidence of headache and vomiting was 7.8 and 17.6%, respectively (Figure 5B), with a statistically significant difference between the two groups (*p* < 0.05). In addition to the increased intracranial pressure due to intracranial occupancy, headache can also be associated with neurological and vascular disorders. Additionally, associated neuropeptide or vasoactive factor release can also result in vomiting in addition to gastrointestinal dysfunction. Consequently, the 469 genes related to neurological and vascular conditions were analyzed.

### Variance Analysis in the TCGA Database

**Figure 6:**
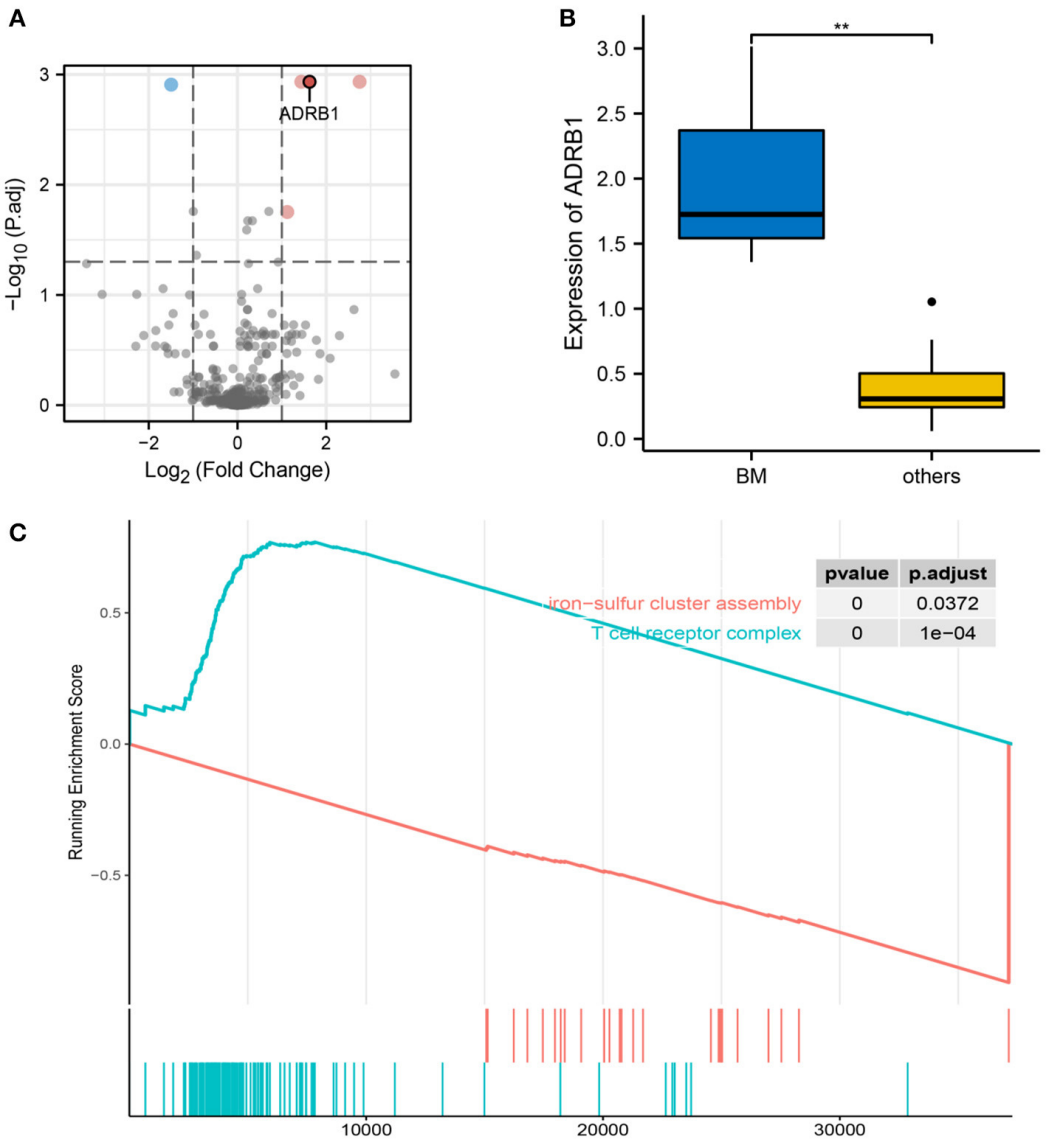
Characteristics of ADRB1. **(A)** Volcano plot showing differentially expressed genes between the brain metastasis and the extracerebral metastasis groups, and *ADRB1* was upregulated in the brain metastasis group. **(B)** Differences in *ADRB1* expression between the brain metastasis and non-brain metastasis groups. **(C)** GSEA showing the upregulation of the T cell receptor complex pathway in the ADRB1-enhanced group and the downregulation of the iron-sulfur cluster assembly pathway.

RNA transcription data were obtained from the TCGA database for three patients with cervical cancer who had brain metastases and 14 patients with cervical cancer who did not have brain metastases. Differential expression analysis was performed on the 469 genes related to neurological function, which were obtained from the literature review and GeneCards database. Five genes (*CHGB, CACNA1B, ADRB1, PLCG1* and *TSPO*) were found to be statistically significant between the two groups (Figure 6A). After ranking the differential genes by significance and investigating the function of each gene, *ADRB1* was identified as a gene that could be involved in nerves and blood vessels. *ADRB1* expression was significantly different in brain metastasis compared to non-brain metastasis groups (Figure 6B). In the tumor brain metastasis group, *ADRB1* expression was significantly higher than at other metastasis-site group (*p* = 0.003). In order to further identify the role of *ADRB1* in cervical cancer brain metastasis, the GSEA analysis of RNA transcription data from patients with cervical cancer revealed that the T cell receptor complex pathway was upregulated, while the iron-sulfur cluster assembly pathway was downregulated (Figure 6C). This suggests the potential role of *ADRB1* in immune regulation and altered mitochondrial respiratory function in response to hypoxia (48).

### Radiomics Features Can Predict *ADRB1* Expression

**Figure 7:**
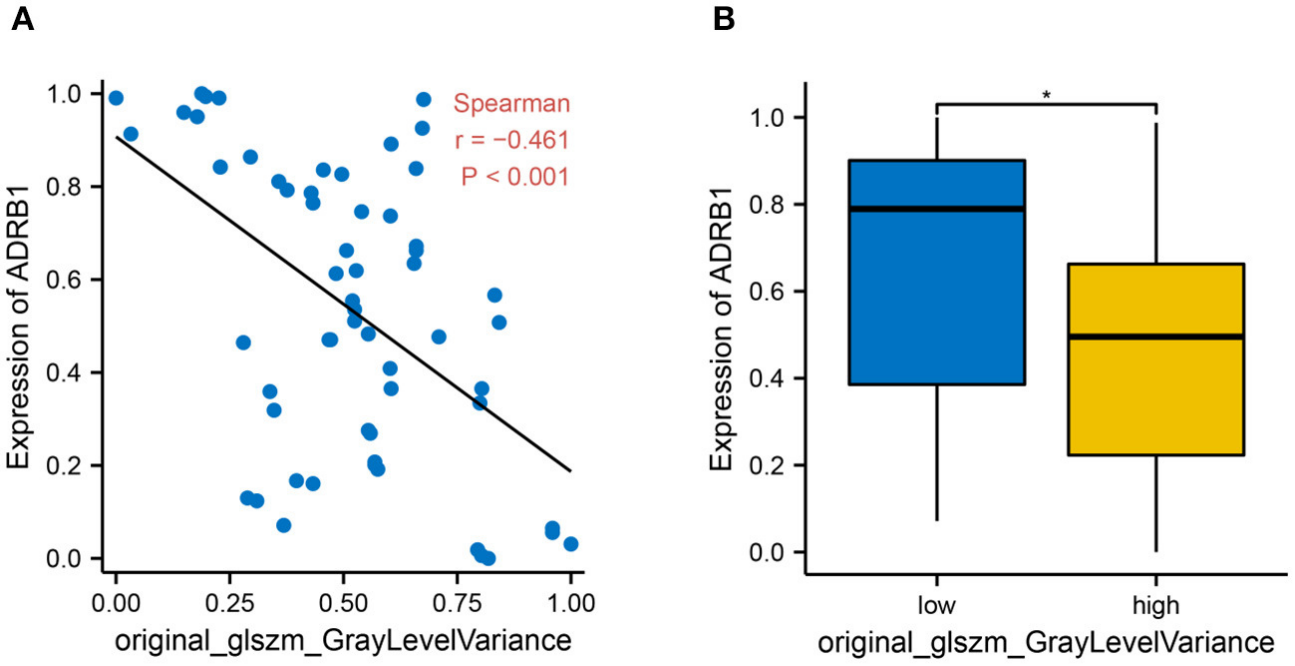
The relationship between histological features and *ADRB1* expression. **(A)** A correlation analysis found a negative relationship between the imaging feature original_glszm_GrayLevelVariance and *ARDB1* expression (Spearman r = −0.46, *P* < 0.001). **(B)** Patients were divided into high Rad-score and low Rad-score groups based on the value of the histological feature original_glszm_GrayLevelVariance; * *p* < 0.05.

A correlation analysis was conducted between original_glszm_GrayLevelVariance and *ADRB1* expression in 60 patients to further examine the predictability of the imaging feature in the constructed model. The correlation analysis revealed a negative correlation between the radiomics feature original_glszm_GrayLevelVariance and *ADRB1* expression (Figure 7A, Spearman r = −0.46, *p* < 0.001). Patients were further divided into high Rad-score and low Rad-score groups according to the value variation of the radiomics feature original_glszm_GrayLevelVariance. The difference in *ADRB1* expression between the high- and low-Rad-score groups is statistically significant (Figure 7B). Expression of ADRB1 was higher in the low-Rad-score group than the high-Rad-score group (*p* = 0.025). In light of this, the radiomics feature original_glszm_GrayLevelVariance in the model is likely to be able to predict *ADRB1* expression and, thereby, brain metastasis in cervical cancer. Hence, *ADRB1* expression is associated with radiomics features and is considered a risk factor for cerebral neurovascular metastasis of cervical cancer.

## Discussion

The clinical and radiomic features of 118 patients with cervical cancer (with or without tumor metastasis) were retrospectively analyzed. Univariate and multivariate logistic regression analyses screened four clinical features (age, stage, N and T) and one radiomics feature (original_glszm_GrayLevelVariance) associated with cervical cancer metastasis. These features were used to construct clinical prediction models. *ADRB1* was found to be related to radiomic features and was considered as a risk factor associated with the risk of cerebral neurovascular metastasis in cervical cancer.

Radiomics is an emerging field that involves the transformation of various types of images into high-dimensional image feature data, providing a more comprehensive description of tumor tissue heterogeneity (49, 50). In a study, the prediction of cervical cancer metastasis was modeled using multiparametric MRI imaging and seven clinical and pathological characteristics, demonstrating that multisequence MRI features serve as non-invasive biomarkers for the preoperative assessment of cervical cancer lymph node status. Furthermore, these characteristics can have an impact on treatment decisions for patients with cervical cancer at an early stage (51). Wu et al. constructed a radiomics model and apparent diffusion coefficients (ADCs) of 189 patients, which was able to predict the development of lymph node metastases in cervical cancer with an AUC of 0.847 (52). Similarly to previous MR-based studies of cervical cancer, this study also observed a better predictability of cervical cancer metastasis using radiomics (53). Based on the original_glszm_GrayLevelVariance feature of enhanced CT images in conjunction with clinical features, the current study developed a clinical prediction model for cervical cancer metastasis. It was demonstrated that the prediction model had good predictive efficacy, a high clinical usefulness and a wide safety range, which successfully differentiated between women with cervical cancer who had metastasized and those who did not. Recent studies have shown that radiomics can be used to investigate the association between histological features and gene mutations by mining high-throughput radiomics features from conventional CT images (54). Similarly, radiomics can also predict genes associated with cervical cancer metastasis.

*ADRB1*, a β1 adrenergic receptor, is a member of the G protein-coupled receptor family, serving as adrenergic receptors in myocardiocytes (55). The β-adrenergic receptor signaling pathway has also been found to be closely associated with tumor proliferation, apoptosis and microenvironment regulation (56–59). According to previous studies, ADRB family genes play a crucial role in regulating the interaction between the sympathetic drive and the immune system (60). According to previous studies, ADRB family genes play a crucial role in regulating the interaction between the sympathetic drive and the immune system. *ADRB1* is involved in the regulation of tumor development in the lungs, breast and prostate (61–63). Furthermore, *ADRB1* genes may act synergistically with *FOXA1* and *ADRB2* genes to drive tumor formation and development (62). It is also closely associated with metastasis and recurrence of oral squamous cell carcinoma (64). Similarly, this study identified *ADRB1* as a potential oncogene involved in the neurovascular metastasis of cervical cancer. Consistent with previous studies, GSEA revealed that the T cell receptor complex pathway was upregulated, whereas the iron-sulfur cluster assembly pathway was downregulated in the *ADRB1* group, indicating the role of *ADRB1* in immune regulation and altered mitochondrial respiratory function in response to hypoxia. Wang et al. observed that the high expression of *ADRB1* could mediate and maintain a normal immune response by promoting the activity of B cells and dendritic cells (61). Immune infiltration contributes significantly to tumor progression (65). Thus, *ADRB1* serves as an immunomodulator in the process of cerebral neurovascular metastasis in cervical cancer.

Lymph node status in early-stage cervical cancer was found to be associated with the age of the patient (66). Ki et al. report the mean age of diagnosis in patients with cervical cancer metastasis as 55 years (67). Advanced age is a remarkable factor affecting the prognosis of patients with stage IV cervical cancer, with patients ≥70 years of age having the worst prognosis (68). Similar to previous studies, the age of patients with cervical cancer in the non-metastatic group was found to be significantly lower than those with metastasis. Moreover, age turned out to be an independent risk factor for cervical cancer metastasis. Additionally, patients with cervical cancer who had tumor diameters >4 cm were significantly more likely to develop metastasis than those who had tumor diameters <4 cm (69). The incidence of cervical cancer metastasis increases with the progression of the tumor stage (70). Similarly, pelvic lymph node metastasis in patients with cervical cancer could lead to a higher rate of distant metastasis (71).

Neurovascular factors are thought to contribute to headache and vomiting. The incidence of headache and vomiting was higher in patients with cervical cancer in the brain metastasis group when compared to those with other sites of metastasis. Further bioinformatic analysis revealed that the expression of neurovascular-related gene *ADRB1* was elevated in the tumor brain metastasis group. However, the current study has certain limitations: (1) The clinical aspect of this study was retrospective, with possible selection bias and a small sample size. Therefore, further multi-center studies should expand the sample size; (2) The risk factors for brain metastasis in cervical cancer screened in this study were based on bioinformatic analyses, which require further clinical validation; (3) RNA transcription data were downloaded from the TCGA database for two patients with brain metastases and 15 patients with extracerebral metastases from cervical cancer, which may be subject to selection bias due to the limited sample size; (4) Additionally, further experiments to validate the function of *ADRB1* in cervical cancer brain metastasis are required.

## Conclusion

Based on four clinical features and one radiomic feature, a nomogram-based cervical cancer metastasis prediction model has been developed. A radiomics feature is considered essential for predicting tumor gene expression. *ADRB1* gene expression was significantly higher in cervical cancer brain metastasis than in other metastasis sites. Therefore, features of radiomics that are related to *ADRB1* may be associated with the risk of cerebral neurovascular metastases in cervical cancer.

## Data Availability Statement

The datasets presented in this study can be found in online repositories. The names of the repository/repositories and accession number(s) can be found in the article/supplementary material.

## Ethics Statement

The studies involving human participants were reviewed and approved by Institutional Ethics Review Board of the Guizhou Provincial People' s Hospital. The patients/participants provided their written informed consent to participate in this study.

## Author Contributions

XZ, SX, and JW reviewed the manuscript, analysis, and analyzed the results. XZ drafted and performed the data curation. All authors read and approved the final manuscript.

## Conflict of Interest

The authors declare that the research was conducted in the absence of any commercial or financial relationships that could be construed as a potential conflict of interest.

## Publisher's Note

All claims expressed in this article are solely those of the authors and do not necessarily represent those of their affiliated organizations, or those of the publisher, the editors and the reviewers. Any product that may be evaluated in this article, or claim that may be made by its manufacturer, is not guaranteed or endorsed by the publisher.
